# Effects of Demographic Identities on Psychosocial Burdens of Patients Living in the United States With Systemic Lupus Erythematosus

**DOI:** 10.7759/cureus.75043

**Published:** 2024-12-03

**Authors:** Kellie N Fusco, Luis C Gonzalez Isoba, Rachel Alef, Angelica Roger, Harvey N Mayrovitz

**Affiliations:** 1 Osteopathic Medicine, Nova Southeastern University Dr. Kiran C. Patel College of Osteopathic Medicine, Davie, USA; 2 Family Medicine, Internal Medicine, Surgery, Pathology, Gynecology, Infectious Disease, Neurology, Orthopedics, and Gastroenterology, Nova Southeastern University Dr. Kiran C. Patel College of Osteopathic Medicine, Fort Lauderdale, USA; 3 Medicine, Nova Southeastern University Dr. Kiran C. Patel College of Osteopathic Medicine, Fort Lauderdale, USA; 4 Medicine, Nova Southeastern University Dr. Kiran C. Patel College of Allopathic Medicine, Davie, USA

**Keywords:** autoimmunity, burdens of sle, demography, financial burdens, lupus, mental health burdens, rheumatology, social burdens, systemic lupus erythematosus

## Abstract

Introduction: Systemic lupus erythematosus (SLE) is a chronic autoimmune disease predominantly affecting women, particularly in African American populations. While its physical health impacts are well-documented, patients also face significant psychosocial burdens, including barriers to healthcare access, financial constraints, mental health challenges, and inadequate social support.

Study goal: This cross-sectional study surveyed 294 SLE patients recruited from Facebook and Reddit social media forums to examine how demographic factors such as age, race/ethnicity, and geographic location influence these burdens.

Results: Findings revealed that although most participants had health insurance and access to rheumatology care, the majority were not able to be evaluated by their specialist until at least a week later after inquiring about an appointment, especially urban respondents. This does not appear to be a major limitation as such appointments may take in some cases months. Limited access to a pharmacy was reported more by respondents who are either rural residents, live in the Midwest region of the United States, or are older than 61 years old. Additionally, SLE is shown to impact patients financially. About 20.1% of the respondents indicated that they are unable to afford their co-pay for all of their monthly medications in general. Indian Asians were noted to pay more out-of-pocket medical expenses compared to other race/ethnicity groups. Respondents older than 61 years old were more likely to spend more than $200 a month on prescriptions for all their medical conditions than their younger counterparts. Regarding mental health, higher rates of anxiety and depression were reported across all groups, especially in a higher percentage of younger respondents under the age of 45. Despite this, there were no major differences in mental health changes between race/ethnicity and geographic location groups. Additionally, most respondents reported having some kind of social support for their SLE diagnosis, notably in the older than 61 age group. Suburban and urban respondents also reported receiving more support than rural ones.

Conclusion: Overall, this study highlights the complex interplay of socioeconomic factors and demographic identities in shaping the experiences of SLE patients, emphasizing the need for healthcare providers to consider these nonphysical aspects when developing treatment plans. Tailored interventions are essential to address the unique needs of diverse patient populations and enhance the management of SLE.

## Introduction

Systemic lupus erythematosus (SLE) is a chronic disease due to excessive production of pathogenic autoantibodies that can affect multiple systems and result in organ damage [1]. It is predominant in females of childbearing age, with greater prevalence in the African American population [2]. In a systemic analysis conducted in 2022 by Tian et al., the incidence of SLE in the United States was estimated to be 12.13 per 100,000 person-years, and the incidence in women was 20.51 per 100,000 person-years [3].

This condition has a wide range of clinical features ranging from mild cutaneous findings and arthralgias to severe medical issues such as renal failure requiring hemodialysis, pulmonary arterial hypertension, cardiac failure, and many other life-threatening conditions [1]. SLE can initially present with multiple and complex clinical manifestations that can mimic other conditions, such as rheumatoid arthritis, Sjogren’s syndrome, systemic sclerosis, dermatomyositis, viral infections (i.e., Parvovirus B19), reaction to vaccines, and neoplasms [4]. This can cause difficulty in reaching the correct diagnosis of SLE in a timely manner [5]. Because of the risk of developing multisystem organ failure, it is imperative for SLE to be correctly diagnosed quickly and managed appropriately.

The prominent issue every SLE patient must face involves their medical condition and physical health, but there are other burdens related to their SLE condition that must be dealt with. These underreported burdens SLE patients must endure involving the areas of accessibility to care [6-13], finance [11-12, 14-15], mental health [16-20], and social support [11,21-23]. The medical literature has shown that SLE carries a considerable burden, leading to a consistently lower quality of life compared to those with other chronic diseases [18,24]. The nature and impacts of these burdens are multifold, and a better understanding of their effects on persons with SLE would aid in laying the groundwork for mitigating these impacts. Specifically, this study investigates the potential role or relationship between demographic identities of age, race/ethnicity, and geographic location on the psychosocial burdens of care accessibility, finance, mental health, and social support on one specific health condition, SLE.

## Materials and methods

Participants

Participants were recruited from two social media forums: “Lupus Cures, Diets, and Natural Remedies Research Group” (on Facebook) and “r/LupusResearch” and “r/Lupus” (on Reddit). This anonymous survey posted on the respective social media forums could be accessed by either scanning a mobile device over the QR code on the marketing flier (Appendix A) or could be accessed by going to the listed website URL. The survey was housed in Research Electronic Data Capture (REDCap), which is a secure web application hosted by NSU KPCOM [25,26]. The study was approved by the Nova Southeastern University Institutional Review Board (IRB Study No. 2024-108-NSU).

The inclusion criteria required participants to be at least 18 years old, have a clinical diagnosis of SLE made by a medical provider, currently live in the United States, be proficient in the English language, have access to a computer or mobile device to be able to access the survey and give implied consent.

The exclusion criteria prohibited participants who were under the age of 18 years, did not have SLE, lived in another country outside of the United States, were not proficient in the English language, did not have access to a computer or mobile device, and did not give implied consent.

All participants answered an online anonymous survey that was split into six sections and would ultimately aim to quantitatively measure the effects of demographic identities on psychosocial burdens associated with SLE. To have been included in this study, participants must have answered ‘yes’ to the survey eligibility questions indicating that they are at least 18 years old, have had a clinical diagnosis of SLE made by a medical provider, and live in the United States. 

Survey 

The online anonymous survey of the present study consisted of six separate sections: Eligibility, Demographics, Accessibility, Financial, Mental Health, and Social Support. The eligibility section included three questions (Appendix B), the demographic section had five questions (Appendix C), accessibility had seven questions (Appendix D), financial had four questions (Appendix E), mental health had four questions (Appendix F), and social support had four questions (Appendix G). In total, participants answered 27 questions. The entire survey took approximately six minutes to complete. Before the beginning of the study, participants were presented with an online informed consent form. If consent was given, they selected the *I agree* button, bringing them to the survey's beginning. Upon completion, participants clicked the submission button and could exit the browser.

The specific questions posed to the subjects are detailed in the Appendices. They do not include questions obtaining information regarding other SLE-related diseases such as fibromyalgia, chronic kidney disease, or lupus nephritis as well as additional general medical comorbidities such as diabetes or hypertension. Additionally, the survey did not ask specifically what SLE medications respondents were actively taking including, but not limited to, hydroxychloroquine, biologics, or steroids. Inquiring if respondents were actively on hemodialysis was not demonstrated as well. Though we appreciate these questions as being important in other contexts, they were not included in our survey due to not being relevant to the specific aim of the present study.

Data collection and analysis

The anonymous data from the survey was collected through the REDCap survey collector. On 03/01/2024, the survey was posted for the first time on Facebook and Reddit and reposted to the same sites on 03/11/2024. The survey was terminated one month after reposting. Data were exported to an Excel sheet for further analysis. Cross-tabulation was used to analyze the demographic identities of age, race/ethnicity, and geographic location with psychosocial burdens, including variables within the main domains of accessibility, financial, mental health, and social support. Data were presented as percentages of responses for each question.

## Results

Sample size

A total of 380 anonymous participants’ responses were initially collected online through RedCap. Of the 380 respondents, 61 did not complete the survey in its entirety and 25 did not meet the eligibility requirements. Therefore, 86 participants were omitted from the analysis, leaving the final sample size to be 294.

Ages and gender

Demographics of age, gender, race/ethnicity, and geographic location were obtained from the final sample of participants. The age ranges of respondents were 18 to 30 years (43.5%), 31 to 45 (38.8%), 46 to 60 years (13.6%), and 61 years old or older (4.1%). The majority of the respondents identified with the female gender (88.1%), with the remaining as male (8.2%) or nonbinary (3.7%).

Race and ethnicity

The sample was predominantly composed of White/Caucasian participants (71.1%), followed by Hispanic participants (10.2%), Black/African American participants (7.1%), East Asian participants (6.1%), participants who identified as Other (3.4%), Indian Asian participants (1%), Pacific Islander participants (0.7%), and Alaska Native participants (0.3%). When prompted, most respondents who selected Other as their race/ethnicity reported being of a mixed race, such as Caucasian and African American.

Geographic location

The survey's geographic location section contained two separate categories of states in the U.S. that participants currently reside in and population density. States were then grouped depending on their region in the country. Participants were found to be spread among five major areas of the United States. These included Southeast (26.5%), West (25.5%), Northeast (18.4%), Midwest (17.7%), and Southwest (11.9%). For population density, 57.8% of respondents reported to be located in a suburban area, 22.8% in an urban area, and 19.4% in a rural area.

Care accessibility findings

Overall, 98.3% of respondents reported holding a health insurance policy, with the majority being employment-based (60.9%). Other types of health insurance noted were Medicaid (10.7%), Other (10%), Medicare (9%), Obamacare (7.3%), and Veterans Affairs (2.1%).

When asked about SLE care, most respondents (79.3%) noted having a rheumatologist within a 20-mile radius, and 92.5% were under the specialist care of a rheumatologist. Of those under rheumatologist care, 68.7% reported being able to obtain an appointment with their rheumatologist within the time frame of some point over a week to three months. Regarding the need to be evaluated in the emergency room and/or urgent care for SLE-associated complaints due to not being able to be seen by a rheumatologist or primary care provider, 60.9% did not need to, 33.7% had to 1 to 3 times, 4.1% for 4 to 6 times, and 1.4% for more than 6 times. For pharmacy accessibility, a large portion (95.9%) was noted to have one within a 10-mile radius. The potential effects of demographic identities on the accessibility of SLE care findings were further analyzed with cross-tabulation. Table 1 shows the breakdown with respect to age.

**Table 1:** Potential effects of age on care accessibility for SLE patients. The table shows the percentage of respondents who answered the associated care accessibility survey questions with a specific answer compared to the rest of their particular age group. ER, emergency room; UC, urgent care; SLE, systemic lupus erythematosus

Topic	Survey question	Answer choices	Ages (years)			
			18-30	31-45	46-60	>61
Health Insurance	Covered under health insurance?	Yes	99.2%	97.4%	97.5%	100%
		No	0.8%	2.6%	2.5%	0%
Rheumatologist	Under the management of a rheumatologist?	Yes	91.4%	93.9%	90%	100%
		No	8.6%	6.1%	10%	0%
Rheumatologist distance	How far away is the closest rheumatologist to where you live?	<5 miles	25%	28.1%	27.5%	16.7%
		5-10 miles	29.7%	27.2%	37.5%	41.7%
		10.1-20 miles	28.1%	20.2%	15%	16.7%
		20.1-30 miles	7.8%	8.8%	7.5%	0%
		>30 miles	9.4%	15.8%	12.5%	25%
Rheumatologist appointment	If you need an appointment with your rheumatologist, how long would it take?	Same day	0%	1.9%	0%	8.3%
		No more than a week	17.1%	13.1%	27.8%	16.7%
		>week, but	33.3%	40.2%	27.8%	25%
		>month, but <3 months	37.6%	31.8%	27.8%	33.3%
		>3 months	12%	13.1%	16.7%	16.7%
ER and/or UC visits	Times in the past 12 months did you have to go to the ER and/or UC for SLE complaints because you could not be seen by a rheumatologist or PCP?	None	54.7%	62.3%	72.5%	75%
		1 to 3 times	38.3%	32.5%	25%	25%
		4 to 6 times	5.5%	3.5%	2.5%	4%
		>6 times	1.6%	1.8%	0%	0%
Pharmacy	A pharmacy within 10 miles?	Yes	96.9%	96.5%	97.5%	75%
		No	3.1%	3.5%	2.5%	25%

The breakdown with respect to race/ethnicity is shown in Table 2.

**Table 2:** Potential effects of race/ethnicity on care accessibility for SLE patients. The table shows the percentage of respondents who answered the associated care accessibility survey questions with a specific answer compared to the rest of their particular race/ethnicity group. ER, emergency room; UC, urgent care; SLE, systemic lupus erythematosus

Topic	Survey question	Answer choices	Race/Ethnicity							
			Alaska Native	Black/African American	East Asian	Hispanic	Indian Asian	Pacific Islander	White/Caucasian	Other
Health Insurance	Covered under health insurance?	Yes	100%	90.5%	94.4%	93.3%	100%	100%	100%	100%
		No	0%	9.5%	5.6%	6.7%	0%	0%	0%	0%
Rheumatologist	Under the management of a rheumatologist?	Yes	100%	95.2%	100%	93.3%	100%	50%	91.4%	100%
		No	0%	4.8%	0%	6.7%	0%	50%	8.6%	0%
Rheumatologist Distance	How far away is the closest rheumatologist to where you live?	< 5 miles	0%	23.8%	38.9%	23.3%	0%	0%	25.8%	40%
		5 to 10 miles	100%	33.3%	33.3%	26.7%	66.7%	0%	30.1%	20%
		10.1 to 20 miles	0%	23.8%	16.7%	26.7%	33.3%	100%	22%	20%
		20.1 to 30 miles	0%	4.8%	5.6%	10%	0%	0%	8.1%	10%
		> 30 miles	0%	14.3%	5.6%	13.3%	0%	0%	13.9%	10%
Rheumatologist Appointment	If you need an appointment with your rheumatologist, how long would it take?	Same day	0%	0%	0%	0%	0%	0%	1.6%	0%
		No more than a week	0%	32%	22.2%	7.1%	33.3%	100%	15.7%	10%
		> week, but < month	0%	45%	33.3%	35.7%	66.7%	0%	33%	50%
		> month, but < 3 months	0%	20%	44.4%	42.9%	0%	0%	35.1%	10%
		> 3 months	100%	0%	0%	14.3%	0%	0%	14.7%	30%
ER and/or UC Visits	Times in the past 12 months did you have to go to the ER and/or UC for SLE complaints because you couldn't be seen by a rheumatologist or PCP?	None	100%	52.4%	83.3%	46.7%	33.3%	100%	62.2%	50%
		1 to 3 times	0%	42.9%	16.7%	46.7%	66.7%	0%	31.6%	50%
		4 to 6 times	0%	0%	0%	3.3%	0%	0%	5.3%	0%
		> 6 times	0%	4.8%	0%	3.3%	0%	0%	1%	0%
Pharmacy	A pharmacy within 10 miles?	Yes	100%	100%	100%	100%	100%	100%	94.7%	90%
		No	0%	0%	0%	0%	0%	0%	5.3%	10%

The breakdown with respect to geographic location is shown in Table 3.

**Table 3:** Potential effects of geographic location on care accessibility for SLE patients. The table shows the percentage of respondents who answered the associated care accessibility survey questions with a specific answer compared to the rest of their geographic location group. ER, emergency room; UC, urgent care; SLE, systemic lupus erythematosus

Topic	Survey question	Answer choices	Geographic location							
			Population density			Region in the United States				
			Rural	Suburban	Urban	Northeast	Southeast	Southwest	Midwest	West
Health Insurance	Covered under health insurance?	Yes	100%	98.8%	95.5%	100%	97.4%	97.1%	98.1%	98.7%
		No	0%	1.2%	4.5%	0%	2.6%	2.9%	1.9%	1.3%
Rheumatologist	Under the management of a rheumatologist?	Yes	89.5%	92.4%	95.5%	96.3%	93.6%	85.6%	96.2%	89.3%
		No	10.5%	7.6%	4.5%	3.7%	6.4%	14.3%	3.8%	10.7%
Rheumatologist Distance	How far away is the closest rheumatologist to where you live?	<5 miles	7%	24.7%	46.3%	31.5%	17.9%	31.4%	28.8%	26.7%
		5-10 miles	19.3%	34.1%	29.9%	22.2%	35.9%	22.9%	28.8%	34.7%
		10.1-20 miles	26.3%	25.3%	13.4%	33.3%	23.1%	22.9%	17.3%	18.7%
		20.1-30 miles	14%	7.1%	4.5%	5.6%	11.5%	8.6%	5.8%	6.7%
		>30 miles	33.3%	8.8%	6%	7.4%	11.5%	14.3%	19.2%	13.3%
Rheumatologist Appointment	If you need an appointment with your rheumatologist, how long would it take?	Same day	0%	1.3%	1.6%	0%	4.1%	0%	0%	0%
		No more than a week	15.7%	16.6%	18.8%	21.2%	17.8%	23.3%	14%	11.9%
		> week, but < month	31.4%	36.3%	34.4%	34.6%	37%	36.7%	38%	29.9%
		> month, but <3 months	36.8%	35%	25%	25.0%	34.2%	30%	30%	44.8%
		>3 months	11.8%	10.8%	20.3%	19.2%	6.8%	10%	18%	13.4%
ER and/or UC Visits	Times in the past 12 months did you have to go to the ER and/or UC for SLE complaints because you couldn't be seen by a rheumatologist or PCP?	None	64.9%	59.4%	61.2%	61.1%	66.7%	48.6%	73.1%	52%
		1-3 times	31.6%	34.7%	32.8%	31.5%	29.5%	42.9%	23.1%	42.7%
		4-6 times	3.5%	4.1%	4.5%	7.4%	2.6%	5.7%	3.8%	2.7%
		>6 times	0%	1.8%	1.5%	0%	1.3%	2.9%	0%	2.7%
Pharmacy	A pharmacy within 10 miles?	Yes	87.7%	98.2%	97%	96.3%	98.7%	97.1%	86.5%	98.7%
		No	12.3%	1.8%	3%	3.7%	1.3%	2.9%	13.5%	1.3%

Finance-related findings

Within the sample, the majority of respondents reported out-of-pocket expenses for office visits, hospital stays, and/or emergency room visits to be less than $5,000 (84%) over the past year. Regarding monthly out-of-pocket expenses for prescription medications, 11.2% spend more than $200 every month, 12.9% $101 to $200, 28.9% $51 to $100, 32% less than $50, and 15% reported none. Of the total respondents, 20.1% indicated that they were unable to afford their monthly medications and 39.5% admitted to ignoring their symptoms and/or withholding taking their medicines on some days because of the worry about the financial burden. The potential effects of demographic identities on the economic burdens experienced by SLE patients were further assessed with cross-tabulation, as seen in the tables. The direct correlation of financial-related burdens with specific health insurance plans was not assessed with this cross-tabulation. Table 4 shows the breakdown of the financial aspect with respect to age.

**Table 4:** Potential effects of age on financial burdens for SLE patients. The table shows the percentage of respondents who answered the associated financial survey questions with a specific answer choice compared to the rest of their particular age group. ER, emergency room; SLE, systemic lupus erythematosus

Topic	Survey question	Answer choices	Age (years)			
			18-30	31-45	46-60	>61
Medical Visits	Amount paid out-of-pocket in the last 12 months for outpatient office visits, inpatient hospital stays, and/or ER visits for SLE problems?	None	14.1%	12.3%	15%	16.7%
		38.3%	44.7%	43%	41.7%
		$1,000-$4,999	26.6%	29.8%	35%	25%
		$5,000-$10,000	16.4%	10.5%	3%	16.7%
		>$10,000	4.7%	2.6%	5%	0%
Prescriptions	Amount paid out-of-pocket monthly for prescriptions?	None	13.3%	14.9%	20%	16.7%
		37.5%	28.1%	28%	25%
		$51-$100	28.9%	32.5%	20%	25%
		$101-$200	10.9%	13.2%	23%	0%
		>$200	9.4%	11.4%	10%	33.3%

Table 5 shows the breakdown of the financial aspect with respect to race/ethnicity.

**Table 5:** Potential effects of race/ethnicity on financial burdens for SLE patients. The table shows the percentage of respondents who answered the associated financial survey questions with a specific answer choice compared to the rest of their particular race/ethnicity group. ER, emergency room; SLE, systemic lupus erythematosus

Topic	Survey Question	Answer Choices	Race/Ethnicity							
			Alaska Native	Black/African American	East Asian	Hispanic	Indian Asian	Pacific Islander	White/Caucasian	Other
Medical Visits	Amount paid out-of-pocket in the last 12 months for outpatient office visits, inpatient hospital stays, and/or ER visits for SLE problems?	None	0%	9.5%	11.1%	30%	0%	0%	11%	40%
		100%	52.4%	61.1%	36.7%	0%	50%	40.2%	30%
		$1,000-$4,999	0%	19%	11.1%	26.7%	0%	50%	33%	10%
		$5,000-$10,000	0%	9.5%	16.7%	6.7%	67%	0%	12.4%	10%
		>$10,000	0%	9.5%	0%	0%	33%	0%	3.3%	10%
Prescriptions	Amount paid out-of-pocket monthly for prescriptions?	None	0%	9.5%	11.1%	33.3%	0%	50%	12.4%	30%
		100%	33.3%	66.7%	36.7%	0%	0%	29.7%	10%
		$51-$100	0%	33.3%	11.1%	13.3%	33%	0%	32.5%	30%
		$101-$200	0%	9.5%	0%	10%	33%	50%	13.9%	20%
		>$200	0%	14.3%	11.1%	6.7%	33%	0%	11.5%	10%

Table 6 shows the breakdown of the financial aspect with respect to geographic location.

**Table 6:** Potential effects of geographic location on financial burdens for SLE patients. The table shows the percentage of respondents who answered the associated financial survey questions with a specific answer choice compared to the rest of their geographic location group. ER, emergency room; SLE, systemic lupus erythematosus

Topic	Survey question	Answer choices	Geographic location							
			Population density			Region in the United States				
			Rural	Suburban	Urban	Northeast	Southeast	Southwest	Midwest	West
Medical Visits	Amount paid out-of-pocket in the last 12 months for outpatient office visits, inpatient hospital stays, and/or ER visits for SLE problems?	None	10.5%	12.9%	17.9%	14.8%	10.3%	17.1%	15.4%	13.3%
		40.4%	41.2%	43.3%	46.3%	46.2%	34.3%	40.4%	37.3%
		$1,000-$4,999	35.1%	29.4%	22.4%	24.1%	21.8%	28.6%	36.5%	34.7%
		$5,000-$10,000	14%	12.4%	10.4%	11.1%	16.7%	17.1%	5.8%	10.7%
		>$10,000	0%	4.1%	6%	3.7%	5.1%	2.9%	1.9%	4%
Prescriptions	Amount paid out-of-pocket monthly for prescriptions?	None	14%	14.1%	17.9%	14.8%	11.5%	14.3%	17.3%	17.3%
		29.8%	31.2%	35.8%	38.9%	33.3%	31.4%	26.9%	29.3%
		$51-$100	28.1%	30%	26.9%	25.9%	32.1%	37.1%	26.9%	25.3%
		$101-$200	19.3%	11.8%	10.4%	13%	7.7%	11.4%	23.1%	12%
		>$200	8.8%	12.9%	9%	7.4%	15.4%	5.7%	5.8%	16%

Mental health findings

Since their diagnosis of SLE, the majority of respondents have experienced increased feelings of both anxiety (80.6%) and depression (71.8%). Regarding substance use, only a small portion of respondents started or increased the use of alcohol (8.8%) or tobacco (7.8%). The potential effects of demographic identities on mental health and substance use were further assessed with cross-tabulation. Table 7 shows the mental health issues broken down with respect to age.

**Table 7:** Potential effects of age on mental health for SLE patients. The table shows the percentage of respondents who answered the associated mental health survey questions with a specific answer choice compared to the rest of their particular age group. SLE, systemic lupus erythematosus

Topic	Survey question	Answer choices	Age (years)			
			18-30	31-45	46-60	>61
Anxiety	Increased anxiety since diagnosis of SLE?	Yes	80%	83.3%	75%	75%
		No	20%	16.7%	25%	25%
Depression	Increased depression since diagnosis of SLE?	Yes	74.2%	71.9%	72.5%	41.7%
		No	25.8%	28.1%	27.5%	58.3%
Tobacco Use	Started or increased tobacco use since diagnosis of SLE?	Yes	8.6%	9.6%	2.5%	0%
		No	91.4%	90.4%	97.5%	100%
Alcohol Use	Started or increased alcohol use since diagnosis of SLE?	Yes	10.9%	8.8%	5%	0%
		No	89.1%	91.2%	95%	100%

Table 8 shows the mental health issue broken down with respect to race/ethnicity.

**Table 8:** Potential effects of race/ethnicity on mental health for SLE patients. The table shows the percentage of respondents who answered the associated mental health survey questions with a specific answer choice compared to the rest of their particular race/ethnicity group. SLE, systemic lupus erythematosus

Topic	Survey question	Answer choices	Race/Ethnicity							
			Alaska Native	Black/African American	East Asian	Hispanic	Indian Asian	Pacific Islander	White/Caucasian	Other
Anxiety	Increased anxiety since diagnosis of SLE?	Yes	0%	85.7%	72.2%	76.7%	100%	100%	82.3%	60%
		No	100%	14.3%	27.8%	23.3%	0%	0%	17.7%	40%
Depression	Increased depression since diagnosis of SLE?	Yes	0%	76.2%	72.2%	70%	100%	100%	71.3%	70%
		No	100%	23.8%	27.8%	30%	0%	0%	28.7%	30%
Tobacco Use	Started or increased tobacco use since diagnosis of SLE?	Yes	0%	0%	5.6%	3.3%	0%	0%	9.1%	20%
		No	100%	100%	94.9%	96.7%	100%	100%	90.9%	80%
Alcohol Use	Started or increased alcohol use since diagnosis of SLE?	Yes	0%	4.8%	0%	3.3%	0%	50%	9.6%	30%
		No	100%	95.2%	100%	96.7%	100%	50%	90.4%	70%

Table 9 shows the mental health issue broken down with respect to geographic location.

**Table 9:** Potential effects of geographic location on mental health for SLE patients. The table shows the percentage of respondents who answered the associated mental health survey questions with a specific answer choice compared to the rest of their geographic location group. SLE, systemic lupus erythematosus

Topic	Survey question	Answer choices	Geographic location							
			Population density			Region in the United States				
			Rural	Suburban	Urban	Northeast	Southeast	Southwest	Midwest	West
Anxiety	Increased anxiety since diagnosis of SLE?	Yes	82.5%	77.6%	86.6%	81.5%	85.9%	77.1%	86.5%	72%
		No	17.5%	22.4%	13.4%	18.5%	14.1%	22.9%	13.5%	28%
Depression	Increased depression since diagnosis of SLE?	Yes	70.2%	70%	77.6%	81.5%	74.4%	77.1%	67.3%	62.7%
		No	29.8%	30%	22.4%	18.5%	25.6%	91.4%	32.7%	37.3%
Tobacco Use	Started or increased tobacco use since diagnosis of SLE?	Yes	12.3%	5.9%	9%	13%	5.1%	8.6%	9.6%	5.3%
		No	87.7%	94.1%	91%	87%	94.9%	91.4%	90.4%	94.7%
Alcohol Use	Started or increased alcohol use since diagnosis of SLE?	Yes	14%	7.6%	7.5%	9.3%	10.3%	0%	13.5%	8%
		No	86%	92.4%	92.5%	90.7%	89.7%	100%	86.5%	92%

Social support findings

Social support was assessed subjectively by respondents noting if they felt support from their friends, family, and/or peers regarding their SLE diagnosis. Different examples of support were given to respondents for reference such as driving them to their doctor appointments, emotional support, etc. Within the sample, only 8.2% of respondents reported having *no support.* The remainder of the respondents answered with the other options of *a little support* (25.9%), *some support but would like more* (32.3%), and *a lot of support* (33.7%). About 63.7% stated that they belong to a social support group, either online or in person. More than half of respondents checked in with their support system either every day (21.4%) or a few times a week (31%), while the remaining checked in less frequently. The potential effects of demographic identities on these social support findings were further assessed with cross-tabulation. Table 10 shows the social support issues broken down with respect to age.

**Table 10:** Potential effects of age on social support for SLE patients. The table shows the percentage of respondents who answered the associated social support survey questions with a specific answer choice compared to the rest of their particular age group. SLE, systemic lupus erythematosus

Topic	Survey question	Answer choices	Age (years)			
			18-30	31-45	46-60	>61
Support Amount	Amount of support felt from your friends, family, and/or peers regarding SLE diagnosis?	None	9.4%	6.1%	12.5%	0%
		A little	20.3%	28.9%	35%	25%
		Some, but would like more	39.1%	33.3%	17.5%	0%
		A lot	31.3%	31.6%	35%	75%
Support Check In	How often do you check in with your support system?	Not applicable	15.6%	17.5%	17.5%	0%
		Every day	18.8%	21.1%	20%	58.3%
		A few times a week	33.6%	29.8%	25%	33.3%
		Once a week	9.4%	7.9%	12.5%	0%
		Every other week	3.9%	7%	5%	0%
		Once a month	10.2%	5.3%	10%	8.3%
		Every few months	7%	9.6%	5%	0%
		Once a year	0.8%	0.9%	2.2%	0%
		More than a year	0.8%	0.9%	2.5%	0%

Table 11 shows the social support issues broken down with respect to race/ethnicity.

**Table 11:** Potential effects of race/ethnicity on social support for SLE patients. The table shows the percentage of respondents who answered the associated social support survey questions with a specific answer choice compared to the rest of their particular race/ethnicity group. SLE, systemic lupus erythematosus

Topic	Survey question	Answer choices	Race/Ethnicity							
			Alaska Native	Black/African American	East Asian	Hispanic	Indian Asian	Pacific Islander	White/Caucasian	Other
Support Amount	Amount of support felt from your friends, family, and/or peers regarding SLE diagnosis?	None	0%	4.8%	16.7%	10%	0%	0%	8.1%	0%
		A little	0%	19.0%	16.7%	20%	33.3%	50%	29.2%	0%
		Some, but would like more	0%	38.1%	22.2%	33.3%	66.7%	50%	30.6%	60%
		A lot	100%	38.1%	44.4%	36.7%	0%	0%	32.1%	40%
Support Check In	How often do you check in with your support system?	Not applicable	100%	9.5%	33.3%	20%	0%	50%	13.9%	20%
		Every day	0%	19%	16.7%	23.3%	0%	0%	22.5%	20%
		A few times a week	0%	14.3%	27.8%	23.3%	66.7%	50%	33.5%	30%
		Once a week	0%	14.3%	11.1%	10%	33.3%	0%	7.7%	10%
		Every other week	0%	9.5%	5.6%	10%	0%	0%	3.8%	10%
		Once a month	0%	4.8%	0%	6.7%	0%	0%	10%	0%
		Every few months	0%	23.8%	0%	6.7%	0%	0%	6.7%	10%
		Once a year	0%	0%	0%	0%	0%	0%	1.4%	0%
		More than a year	0%	4.8%	5.6%	0%	0%	0%	0.5%	0%

Table 12 shows the social support issues broken down with respect to geographic location.

**Table 12:** Potential effects of geographic location on social support for SLE patients. The table shows the percentage of respondents who answered the associated social support survey questions with a specific answer choice compared to the rest of their geographic location group. SLE, systemic lupus erythematosus

Topic	Survey question	Answer choices	Geographic location							
			Population Density			Region in the United States				
			Rural	Suburban	Urban	Northeast	Southeast	Southwest	Midwest	West
Support Amount	Amount of support felt from your friends, family, and/or peers regarding SLE diagnosis?	None	9%	8.2%	7%	13%	7.7%	8.6%	3.8%	8%
		A little	23.9%	24.1%	33.3%	24.1%	17.9%	31.4%	34.6%	26.7%
		Some, but would like more	32.8%	29.4%	40.4%	35.2%	41%	28.6%	26.9%	26.7%
		A lot	19.4%	38.2%	36.8%	27.8%	33.3%	31.4%	34.6%	38.7%
Support Check In	How often do you check in with your support system?	Not applicable	13.4%	12.9%	28.1%	27.8%	16.7%	8.6%	9.6%	14.7%
		Every day	16.4%	23.5%	21.1%	11.1%	32.1%	25.7%	23.1%	14.7%
		A few times a week	28.4%	29.4%	38.6%	29.6%	21.8%	37.1%	32.7%	37.3%
		Once a week	3%	10%	12.3%	14.8%	5.1%	11.4%	9.6%	6.7%
		Every other week	4.5%	5.3%	5.3%	1.9%	1.3%	8.6%	3.8%	10.7%
		Once a month	9%	8.2%	7%	9.3%	7.7%	2.9%	9.6%	9.3%
		Every few months	9%	8.2%	3.5%	3.7%	14.1%	2.9%	5.8%	6.7%
		Once a year	0%	1.2%	1.8%	0%	1.3%	2.9%	1.9%	0%
		More than a year	1.5%	1.2%	0%	1.9%	0%	0%	3.8%	0%

**Figure 1:**
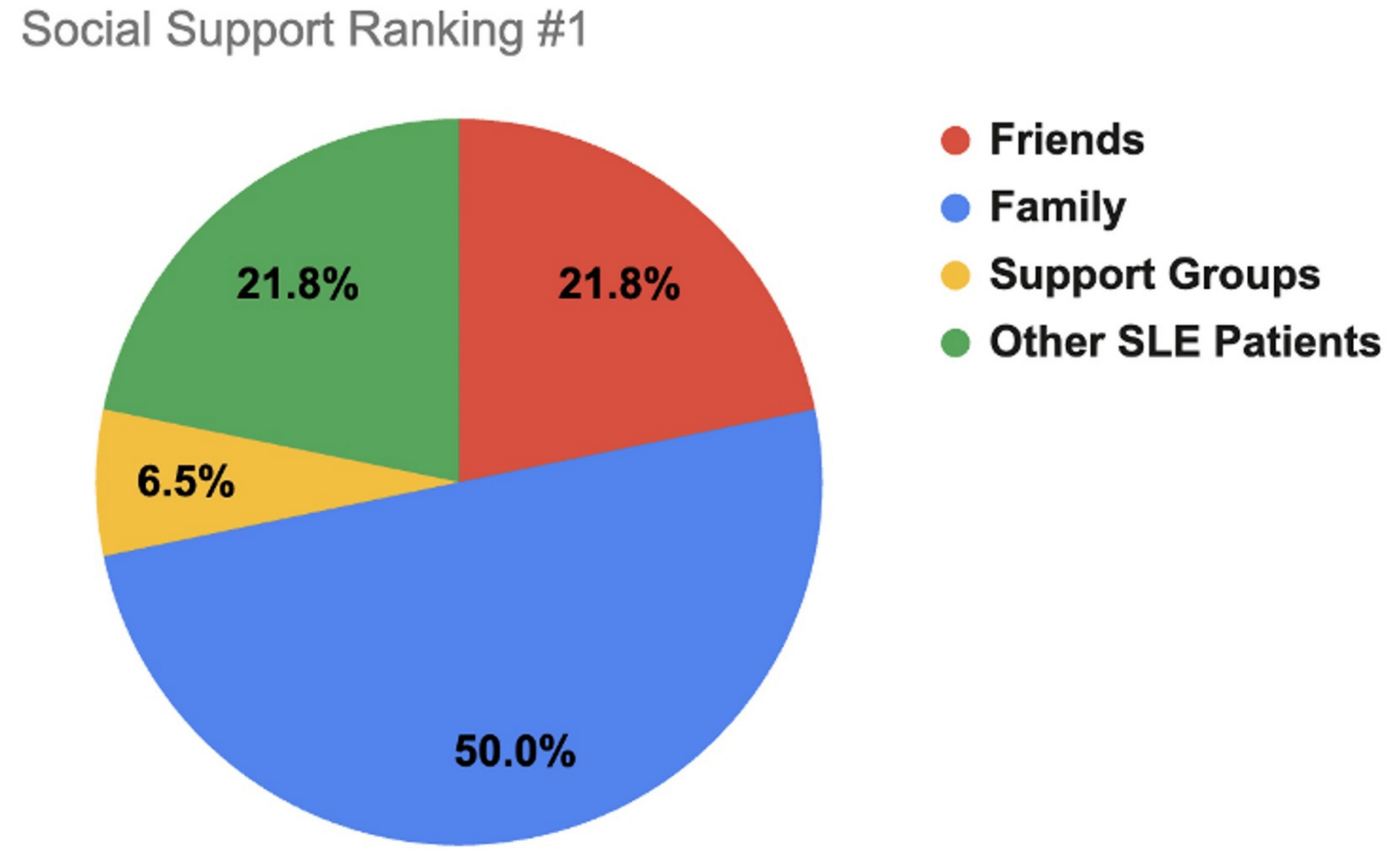
Social support ranking #1 (most support). This figure shows how SLE respondents rated which support system gives them the most amount of support (1 out of 4), with 1 indicating the most support and 4 the least support. Friends is indicated by red coloring, family by blue, support groups by yellow, and other SLE patients by green. SLE, systemic lupus erythematosus

**Figure 2:**
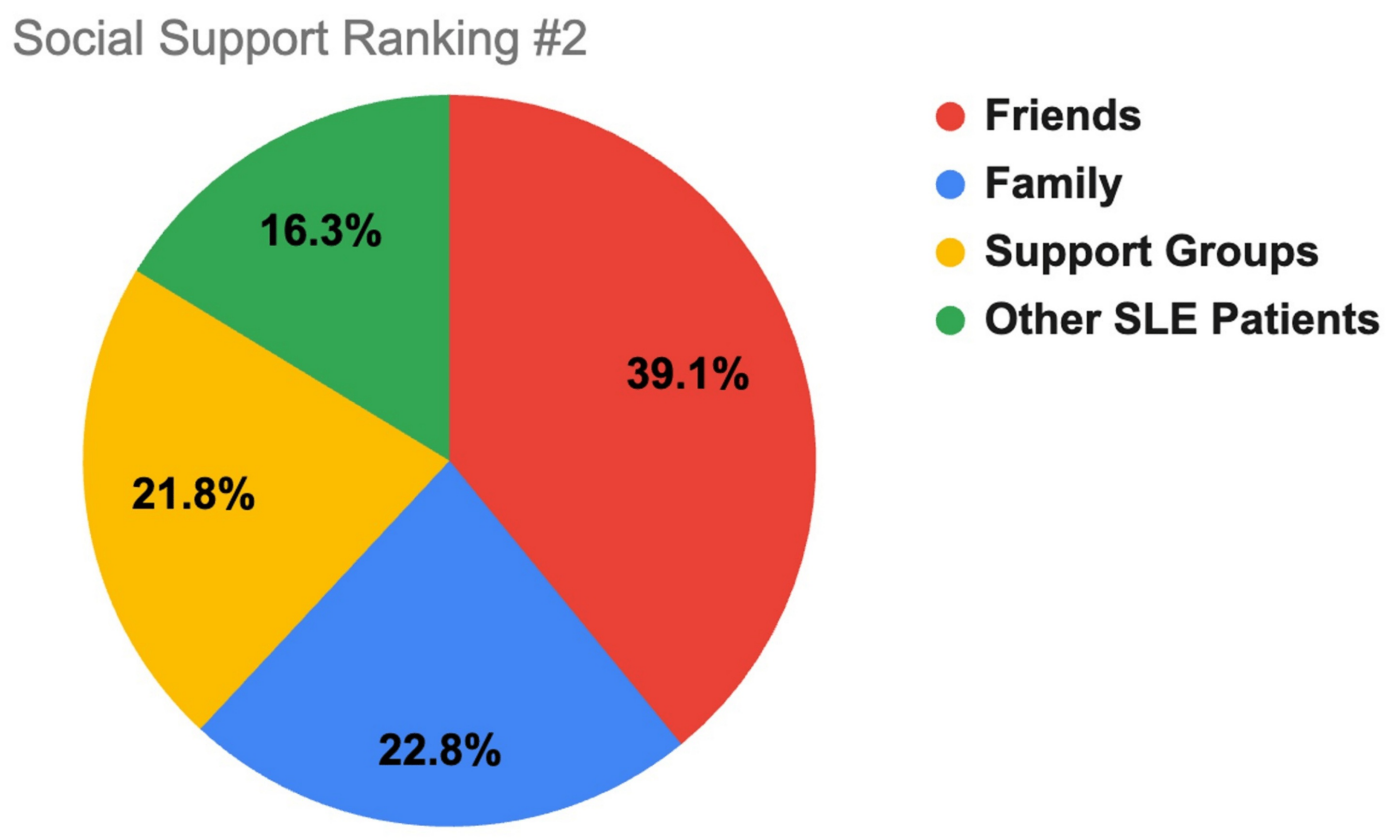
Social support ranking #2. This figure shows how SLE respondents rated which support system gives them a moderate amount of support (2 out of 4), with 1 indicating the most support and 4 the least support. Friends is indicated by red coloring, family by blue, support groups by yellow, and other SLE patients by green. SLE, systemic lupus erythematosus

**Figure 3:**
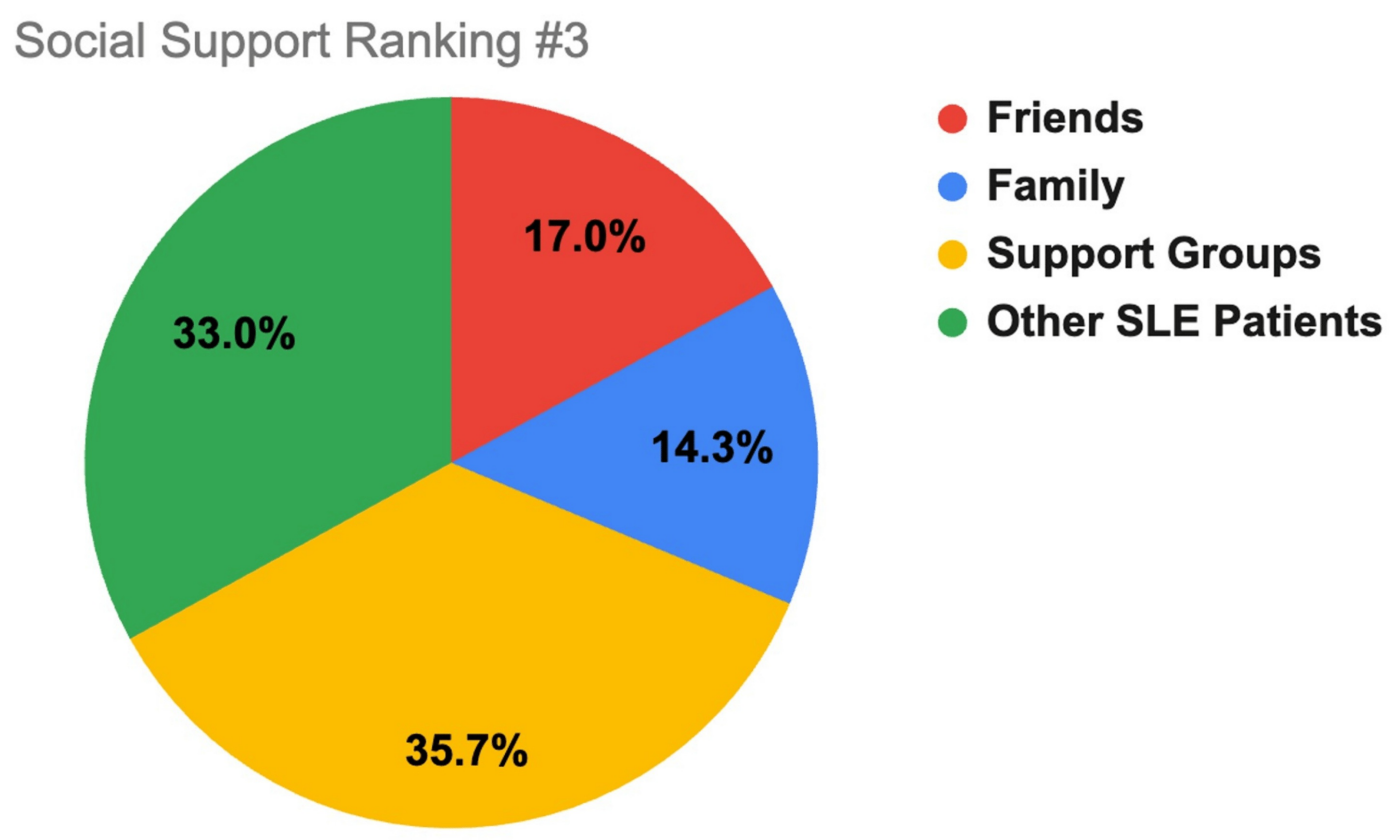
Social support ranking #3. This figure shows how SLE respondents rated which support system gives them a mild amount of support (3 out of 4)., with 1 indicating the most support and 4 the least support. Friends is indicated by red coloring, family by blue, support groups by yellow, and other SLE patients by green. SLE, systemic lupus erythematosus

**Figure 4:**
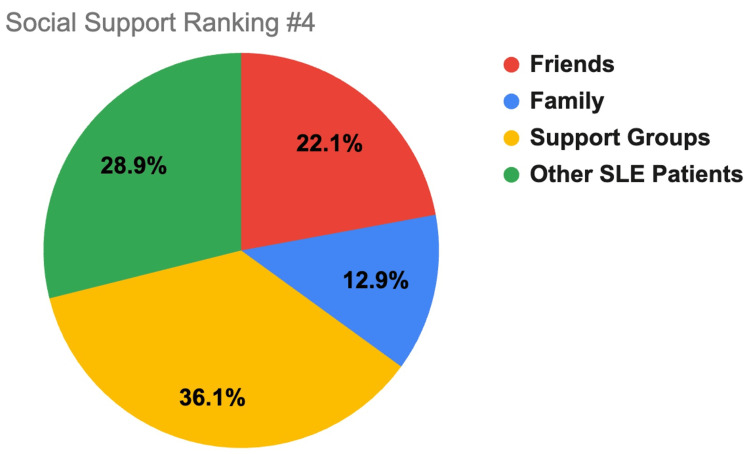
Social support ranking #4. This figure shows how SLE respondents rated which support system gives them the least amount of support (4 out of 4), with 1 indicating the most support and 4 the least support. Friends is indicated by red coloring, family by blue, support groups by yellow, and other SLE patients by green. SLE, systemic lupus erythematosus

Participants ranked where they found the most support to the least support between the different support systems of family, friends, support groups, and other SLE patients. Ranking selection consisted of 1 (most support), 2, 3, and 4 (least support). These findings are displayed in Figures 1-4.

## Discussion

Care accessibility 

Timely and appropriate access to high-quality healthcare, especially to a specialist, is the basis of SLE management [6]. Accessibility in healthcare can include access to health insurance and appropriate therapies, as well as the ability to be evaluated by a nearby medical provider in a timely manner. According to the 2019 Addressing Lupus Pillar of Health Advancement project, the top three barriers to access to care in SLE patients were social health factors in low socioeconomic backgrounds, limited access to SLE specialists, and lack of insurance coverage or high costs of medications [7]. Race and ethnicity can enhance this barrier slightly as seen in our study by there being less health insurance coverage in African American (90.5%), Hispanic (93.3%), and East Asian (94.4%) populations compared to the other race/ethnicity groups with 100% of respondents reporting coverage.

To amplify this ongoing issue in healthcare accessibility, it has been reported that there is a current shortage of rheumatologists, which has been noted to worsen over the next decade, resulting in limited availability of specialists for SLE patients [8]. In our study, almost all of the respondents were under the care of a rheumatologist, but the majority were unable to be evaluated by their specialist until at least a week later (81.9%) after inquiring about an appointment. This does not appear to be a major limitation as such appointments may take in some cases months. Urban residents were more likely (20.3%) than rural and suburban counterparts, 11.8% and 10.8% respectively, to wait more than three months for their appointment. This may be due to the highly dense population of people in their area and a shortage of rheumatologists, causing limited appointment availability. Interestingly, though, more older respondents (older than 61 years) reported the ability to be seen on the same day. Even though in this group, more than one-third of respondents note taking a month to be seen by a rheumatologist, 8.3% can be seen on the same day, while only 1.9% of respondents 31 to 45 years old can be seen within the same day. Additionally, no respondents aged 18 to 30 or 46 to 60 years old even reported the ability to have a same-day appointment. The incapability of the majority of SLE patients to seek specialist care immediately when an issue arises magnifies the issue of timely appropriate care. This leads to some SLE patients seeking evaluation from less specialized providers in the emergency room and/or urgent care. Specifically, in our study, the 18 to 31-year-old age group was required to do so more compared to the other age groups.

Insurance coverage can also be a major barrier to receiving appropriate management for chronic diseases. It can determine what medical provider patients can see and provide financial coverage for these visits and medications. While most of the respondents in our study have both health insurance and specific rheumatology care, there is about 6% of those who have health insurance that are not under the care of a rheumatologist. This may be due to the lack of coverage in specialty care from some insurance companies.

Specifically, patients with SLE under Medicaid coverage tend to face additional barriers to healthcare access. Medicaid is a public health insurance program that provides health care to some of the most vulnerable populations in the U.S. [6]. Despite only 10.7% of respondents in our study being under Medicaid, a large number of SLE patients receive their health insurance coverage through this program [6]. One study had shown that Medicaid SLE patients reported the need to travel longer distances to be seen by their specialists, and this was even more emphasized in patients who live in rural areas [9]. A decrease in rheumatologist care in rural populations compared to suburban and urban was also seen in our study and residents in the Southwest region of the U.S.

In addition to insurance coverage, travel burden has been noted to inhibit accessibility of healthcare in SLE patients. This can lead to missed appointments and medication nonadherence, therefore impacting appropriate disease management [10]. The majority of the rural respondents in our study (33.3%) reported having to travel over 30 miles to be evaluated by their rheumatologist, compared to urban (6%) and suburban (8.8%) respondents. Rural residence has also been associated with worsened SLE manifestations including renal disease, cutaneous features, mental health problems, and overall higher disease activity. This has been hypothesized to be due to rural SLE populations having poorer access to specialized care [11]. Additionally, more of our respondents in the older than 61 years age group (25%) reported traveling greater than 30 miles to their specialist compared to other age groups.

Prescription access can be limited to insurance coverage, medication availability, and financial reasons. A Michigan study noted that Medicaid or no insurance SLE patients were 3 to 7 times more likely to report being unable to obtain their medications [12]. The location of a pharmacy can also limit prescription access since rural areas tend to have pharmacies located farther away, as seen in our study. More of our respondents in the Midwest region of the U.S. (13.5%), as well as ages older than 61 years old (25%), also reported traveling a farther distance to their pharmacy. Additionally, access to therapeutic agents that have been proven to improve disease activity and minimize the progression of organ damage, such as biological agents, is limited in some regions [13]. This demonstrates that accessibility to care is a significant element to consider in successfully managing a chronic disease and understanding social factors' influence on health.

Finance

It is known that individuals’ financial situations play a significant role in how their SLE diagnosis is managed. Most participants in this project’s sample; across all ages, races ethnicities, and geographic locations; reported some financial burden caused by their diagnosis. Given the high costs that come with frequent hospitalizations, outpatient doctors’ appointments, and expensive medication regimens, it’s not surprising that patients diagnosed with SLE have much greater all-cause direct medical costs when compared to non-SLE patients [14].

Structured interviews from patients in the Michigan Lupus Epidemiology and Surveillance (MILES) cohort found that access to prescribed medications was troublesome for both SLE patients and control patients. Still, SLE patients were twice as likely as controls to report cost-related prescription non-adherence to save money. Non-adherence was defined as skipping doses, taking less medicine, and delaying filling prescriptions. They were also more likely to request lower-cost alternatives from their doctors [12]. Our study gives further evidence of this issue. When asked about the impact of their financial situation on their SLE treatment, 20.1% of the respondents indicated that they are unable to afford their co-pay for all of their monthly medications in general, and 39.5% admit to ignoring their symptoms and/or withholding taking their medications on some days because of the worry about budgetary challenges. These findings do not distinguish those on Medicaid, Medicare, or private insurance.

Because of the financial burden that comes with this condition, patients of lower socioeconomic status may be at risk of not being adequately managed and for their disease to worsen. A longitudinal population-based study in Canada, a country with a universal healthcare system, found that low socioeconomic status at SLE diagnosis was associated with significantly greater direct medical costs for managing SLE and associated complications compared to high socioeconomic status patients [14]. According to one report [15], because low-income patients have other, more pertinent issues to worry about, such as housing and food insecurities, some patients are only able to manage their SLE during flares. By this, the authors interpreted the respondents as only being evaluated by the appropriate specialists once symptoms or disease condition progresses too severely.

There has also been an association between lower income level and poverty and multiple adverse outcomes in SLE, some of which are increased disease activity, organ damage, mortality, depression, work loss or disability, and decreased physical functioning and quality of life [11]. Our study noted a difference in the out-of-pocket expenses in Indian Asians, in which more of this population spent more than $10,000 in medical visits (33%) and more than $200 a month for prescriptions (33%) compared to the other groups who had less than 10% and 15% respectively. Overall, all Indian Asians in our study reported spending more than $50 a month on prescriptions while the majority of the other groups spent less than $50. Additionally, regarding age, respondents older than 61 years old were more likely to spend more than $200 a month on prescriptions (33.3%) compared to other age groups (9.4% to 11.4%). This could be due to the older population having more medical conditions, thus requiring more medications for treatment. This survey, however, did not reveal any significant difference in the out-of-pocket expenses for medical visits or prescriptions when separating groups by geographic location. Future research should be conducted that can identify how lower-income patients might be suffering the consequences of untreated SLE due to their inability to finance their disease management.

Mental health

Patients diagnosed with SLE are at an increased risk of having associated deterioration in their mental health status. This study indicates that most respondents self-reported both anxiety (80.6%) and depression (71.8%). Findings demonstrated anxiety and depression impacted patients regardless of age; interestingly, however, people aged 61 or older (58.3%) reported not having any increase in feelings of depression compared to their younger counterparts. This is something that should be further studied; there is a chance, however, that this is due to a smaller sample size of patients aged older than 61 years old (12 patients). It is also possible that older patients already had a previous diagnosis of their depressive symptoms, or this could have been due to some other confounding variables. Despite the smaller number of respondents who self-identified as either Indian Asian or Pacific Islander, every one of them reported increased levels of both anxiety and depression. Regardless of race/ethnicity or geographic location, most patients tended to have increased feelings of depression and anxiety before their diagnosis.

These results, while providing extra demographic information, support many of the previous, broader studies that have also shown a similar deterioration in SLE patient mental health. In a study conducted by Duca et al., the impact of patients’ mental health was compared to their ability to function. Data was collected from 69 SLE patients in an outpatient clinic in Romania. These patients were not undergoing any medical or therapeutic treatment for anxiety or depression. It was found that 85% of patients screened positive for depression (using the 17-Hamilton Depression Rating Scale [HAM-D17]), whereas 61.29% of patients screened positive for anxiety (using the Hamilton Anxiety Rating Scale [HAM-A]) [16].

In a Russian study, Lisitsyna et al. found that in 115 SLE patients, 66% of patients experienced combined anxiety and depression, 40% of patients experienced depressive episodes, and 10% of patients experienced anxiety [17]. Similar findings were found in a case-control study by Primavera et al. looking specifically at depressive episodes in patients. Twenty-nine female SLE patients from the Lupus Clinic of Cagliari were compared to 116 control patients from the general Italian population. Using the Patient Health Questionnaire 9 (PHQ-9) to identify depressive episodes, 34.7% of SLE patients screened positive. While this study does not demonstrate causal effects, it does show a statistically significant difference in association between depressive episodes in patients with SLE in comparison to the general population [18]. In a study analyzing 301 American women with SLE by a telephone composite international diagnostic interview (CIDI), 47% of respondents met the criteria for major depressive disorder (MDD) and 4% met the criteria for generalized anxiety disorder (this data does not include the study’s findings of other mood and anxiety disorders) [19]. Findings from this study provide further evidence to previously conducted studies indicating an association between mental health illnesses, especially anxiety and depression, in patients diagnosed with SLE.

Interestingly, there are minimal studies analyzing the association between SLE and substance abuse (abuse of alcohol, nicotine products, or illicit drugs) resulting from a possible complication of associated mental illness. In a study conducted by Weelden et al., 34 adolescents (age 10-19 years) from Brazil’s Hospital das Clínicas da Faculdade de Medicina da Universidade de São Paulo Pediatric Rheumatology Unit were compared to 35 control patients without SLE. The subjects were given a questionnaire investigating their use of substances (alcohol, cigarettes, illicit drugs). Results showed no statistically significant difference in substance use between subjects with SLE (38% of subjects) and subjects without SLE (48% of subjects) [20]. Similarly to Weelden’s study, this current study demonstrated most patients with SLE did not start or increase their use of tobacco (92.2%) or alcohol (91.2%). However, those who did were primarily younger than 45 years for both substances and were more likely to reside in rural areas for alcohol consumption. Aside from these two studies, data is limited regarding substance abuse in SLE patients. It is important, however, to continue analyzing substance use rates in adults with SLE, as this is the typical age of onset in the general population. With high rates of associated anxiety and depression reported in adults with this chronic illness, there may also be an association with higher substance use in comparison to the general population.

Social support

A major determinant of SLE patient mental health is their level of social support. This study demonstrates that most respondents had at least some level of support (91.9%) with most reported support coming from family (50%), most belonging to support groups (67.70%), and most patients checking in with their support systems at least once a week (61.2%). Compared to other age groups, respondents older than 61 years old were more likely to receive a lot of support (75%) and check in more frequently with their support system (58.3% reported checking in daily) than their younger counterparts (less than/equal to 35% of participants younger than 61 reported feeling a lot of support and 21.1% or less of participants younger than 61 reported checking in daily). Additionally, suburban (38.2% *a lot*) and urban (36.8% *a lot*) groups reported receiving more support than rural (19.2% *a lot*) respondents, which may be due to greater population density allowing more social support options.

Social support in the form of marriage or living with another person, for example, has been linked to overall better health outcomes in SLE [21]. Recently in 2022, a study was done that questioned the association between social support and quality of life among Chinese women with SLE. It was found that having strong social support, whether it be family, friends, or support groups, increased quality of life. This was possibly due to their access to help when dealing with the difficulties associated with SLE [22]. This is a beneficial finding, especially for the patients in this study, as most of them had at least some form of support.

In another study, the relationship between social support and uncertainty regarding SLE in SLE patients was evaluated. The study details how the complexity of SLE, with its chronic nature and alternating periods of flare-ups and remission, makes it difficult for patients to truly understand their disease, the disease process, and their prognosis, and identify notable health events about their disease. Social support rating scales were completed by 200 SLE patients in a hospital in Shaanxi to assess their level of subjective support, objective support, and support availability regarding their diagnosis, and compare said scores to that of non-SLE, healthy, Chinese people. The study concluded that the hospitalized SLE patients all had lower social support scores when compared to the healthy Chinese population. There was a negative correlation between illness uncertainty and support availability, which indicates that these hospitalized patients were receiving less social support. Increasing patient social support could potentially help lower any level of uncertainty among SLE patients [23].

In 2019, a survey-based study of 246 SLE patients was conducted that further investigated the effect of social support on the overall mental health of SLE patients. In this study, unmarried patients, under the age of 18, or unemployed had lower levels of social support, which subsequently led to higher rates of anxiety and depression being linked to worse health outcomes in SLE patients [11]. Luckily, in this study, most patients, regardless of demographic identity, had some sort of social support. However, most still suffered from increased feelings of depression and anxiety since their diagnosis. These nonphysical burdens that SLE patients experience should continue to be continuously studied in hopes of bringing further awareness to healthcare and general public communities.

Study limitations

While results from this study have given significant insight into the impact of varying demographic identities on psychosocial burdens in SLE patients, this study is not without limitations. Primarily, 294 participants’ data were used in this investigation; this is a smaller sample size than what would have been ideal. Notably, throughout the study, due to specifically collected patient population data (i.e., Indian Asian, Pacific Islander, Alaska Native, respondents 61 or older, etc.) being smaller, some data may be skewed due to the low sample size available. In future studies, if studying particular demographic identities, it may be beneficial to make sure 100-200+ participants come from each identity, as this may make data less skewed and more accurate. Additionally, considering the survey provided was completely anonymous, respondents may have reported inaccurate information. This would be difficult to fact-check since the privacy of respondents is important, but it is something to consider when analyzing data. Finally, it is possible that respondents filled out the survey more than once. While unlikely, it would be good to cap a future study like this to one response per respondent. With these limitations, however, this study is still beneficial in showing trends that demographic identities impact the psychosocial burdens of these patients. Further research should be considered on the health of those with varying identities diagnosed with SLE.

## Conclusions

Patients with SLE may face various psychosocial burdens and struggle with accessibility to healthcare, financial strains, mental health issues, and lack of social support. The data obtained in the present study are consistent with the view that many patients face these barriers. Furthermore, the findings also demonstrate examples in which the diversity of patient demographic identities could contribute to the severity of these burdens. Providers, especially rheumatologists and primary care providers, should keep these nonphysical patient burdens in mind and be aware that patient age, race/ethnicity, and geographic location might influence patient outcome, which is an important consideration to keep in mind for patient treatment plans. It would be beneficial for future studies to further research the correlation between demographic identities and psychosocial burdens in SLE patients to serve various patient populations better. If, for example, a specific patient population with the diagnosis of SLE is particularly having difficulty with their SLE management, obtaining a larger sample size of patients with a more specific demographic identity could be beneficial to understand what this patient population needs to enhance and supplement treatment. Overall, demographic identities do seem to impact psychosocial burdens in patients with SLE, and this is something that patient providers should always consider.

## Figures and Tables

**Figure 5:**
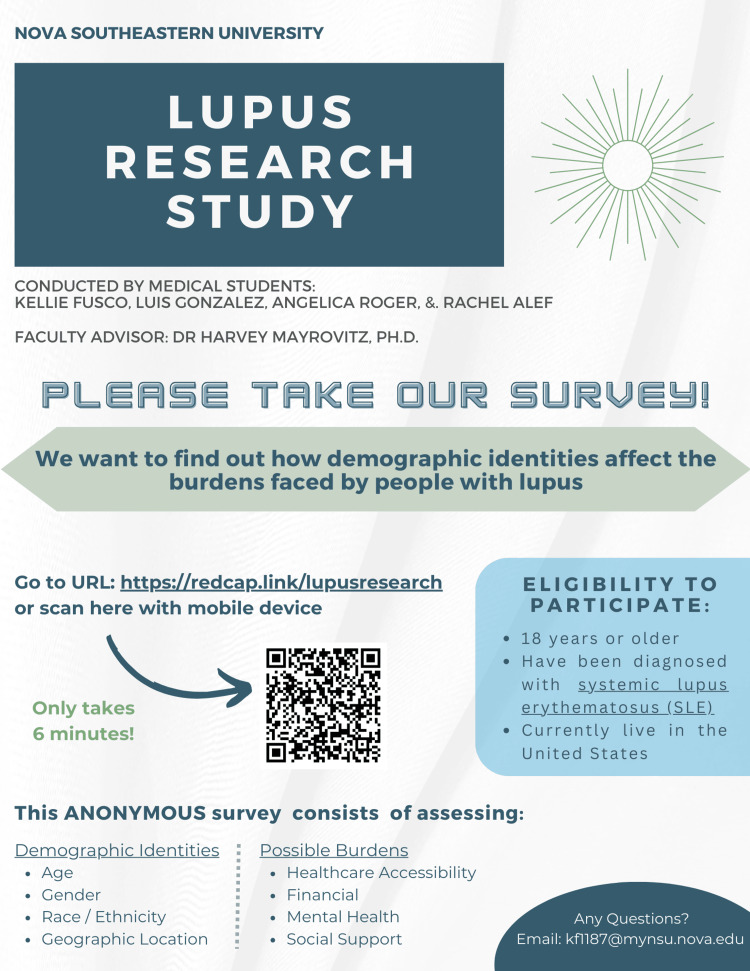
Social media flyer.

## Appendices

 Appendix A

Appendix B

**Table 13:** Survey eligibility questions.

Question	Answer Choices
Section 1: Eligibility	
Have you been diagnosed with systemic lupus erythematosus?	Yes, No
Are you over the age of 18 years old?	Yes, No
Do you currently live in the United States?	Yes, No

Appendix C 

**Table 14:** Survey demographic questions.

Question	Answer Choices
Section 2: Demographics	
How old are you? Please round to the nearest whole number.	18 to 30 years old, 21 to 45 years old, 46 to 60 years old, 61 years old or older
What gender do you identify best with?	Male, Female, Nonbinary, Prefer to self-describe (free text)
What race/ethnicity best describes you?	Alaska Native, Black/African American, East Asian (e.g. Chinese, Japanese, Korean), Hispanic, Indian Asian, Native American, Pacific Islander, White/Caucasian, Other (free text)
What state in the United States do you currently live in?	Alabama, Alaska, Arizona, Arkansas, California, Colorado, Connecticut, Delaware, Florida, Georgia, Hawaii, Idaho, Illinois, Indiana, Iowa, Kansas, Kentucky, Louisiana, Maine, Maryland, Massachusetts, Michigan, Minnesota, Mississippi, Missouri, Montana, Nebraska, Nevada, New Hampshire, New Jersey, New Mexico, New York, North Carolina, North Dakota, Ohio, Oklahoma, Oregon, Pennsylvania, Rhode Island, South Carolina, South Dakota, Tennessee, Texas, Utah, Vermont, Virginia, Washington, West Virginia, Wisconsin, Wyoming
How would you describe the geographic location that you currently live in?	Urban, Suburban, Rural

Appendix D 

**Table 15:** Survey accessibility questions.

Question	Answer Choices
Section 3: Accessibility	
Are you currently covered under health insurance?	Yes, No
What is your source of health insurance coverage?	Employment based, Medicare, Medicaid, Veterans Affairs, Obamacare, Other (free text)
Are you currently under the management of a rheumatologist?	Yes, No
How far away is the closest rheumatologist to where you live?	Under 5 miles, 5 to 10 miles, 10.1 to 20 miles, 20.1 to 30 miles, Greater than 30 miles
If you needed an appointment with your rheumatologist, how long would it take?	Same day, No more than a week, More than a week but less than a month, More than a month but less than 3 months, More than 3 months
How many times in the past 12 months did you have to go to the emergency room and/or urgent care for SLE associated complaints because you were not able to be seen by your rheumatologist or primary care provider?	None, 1 to 3 times, 4 to 6 times, More than 6 times
Is there a pharmacy within 10 miles that you can or do use to obtain medications to treat your SLE?	Yes, No

Appendix E 

**Table 16:** Survey financial questions.

Question	Answer Choices
Section 4: Financial	
In total, how much did you pay out-of-pocket in the last 12 months for outpatient office visits, inpatient hospital stays, and/or emergency room visits for SLE associated problems?	None, Less than $1,000, $1,000 to $4,900, $5,000 to $10,000, More than $10,000
On average, how much do you pay out-of-pocket monthly for prescriptions?	None, Less than $50, $51 to $100, $101 to $200, More than $200
Are you able to obtain the medications that are being prescribed to you without significant financial burden?	Yes, No
Have you ever ignored your symptoms and/or ever withheld taking your medications on some days because you were worried about financial burden?	Yes, No

Appendix F 

**Table 17:** Survey mental health questions.

Question	Answer Choices
Section 5: Mental Health	
Since your diagnosis of SLE, do you have increased feelings of anxiety that were not there prior to your diagnosis?	Yes, No
Since your diagnosis of SLE, do you have increased feelings of depression that were not there prior to your diagnosis?	Yes, No
Since your diagnosis of SLE, have you either started or increased the use of tobacco?	Yes, No
Since your diagnosis of SLE, have you either started or increased the use of alcohol?	Yes, No

Appendix G 

**Table 18:** Survey social support questions.

Question	Answer Choices
Section 6: Social Support	
How much support do you feel you have from your friends, family, and/or peers in regard to your SLE diagnosis (e.g. driving you to doctor appointments, emotional support, etc.)?	No support, A little support, Some support but would like more, A lot of support
Please RANK where you feel the most support from most supportive (1) to least supportive (4). Numerical rank choices can only be used ONCE for all four items.	
Do you belong to any social groups for SLE, either online or in person?	Yes, No
How often do you check in with your support system?	Not applicable, Every day, A few times a week, Once a week, Every other week, Once a month, Every few months, Once a year, More than a year
